# Evaluation of New Antimicrobial Agents Based on *tris*(1*H*-Indol-3-yl)methylium Salts: Activity, Toxicity, Suppression of Experimental Sepsis in Mice

**DOI:** 10.3390/ph15020118

**Published:** 2022-01-19

**Authors:** Alexey S. Trenin, Elena B. Isakova, Michael I. Treshchalin, Vasilisa A. Polozkova, Elena P. Mirchink, Alexey A. Panov, Alexander Y. Simonov, Olga P. Bychkova, Victor V. Tatarskiy, Sergey N. Lavrenov

**Affiliations:** 1 Gause Institute of New Antibiotics, 11 B. Pirogovskaya Street, 119021 Moscow, Russia; as-trenin@mail.ru (A.S.T.); ebisakova@yandex.ru (E.B.I.); funky@beatween.ru (M.I.T.); vasilisa2006@gmail.com (V.A.P.); kkna5rf045ex@mail.ru (E.P.M.); simonov-live@inbox.ru (A.Y.S.); olrika@mail.ru (O.P.B.); satory@mail.ru (S.N.L.); 2 Department of Functional Nanosystems and High-Temperature Materials, National University of Science and Technology MISiS, 4 Leninsky Avenue, 119049 Moscow, Russia; tatarskii@gmail.com; 3 Institute of Gene Biology, Russian Academy of Sciences (RAS), Vavilova Street, 34/5, 119334 Moscow, Russia

**Keywords:** *tris*(1*H*-indol-3-yl)methylium, turbomycin, overcoming of drug resistance, toxicity in vitro and in vivo, staphylococcal sepsis

## Abstract

The antimicrobial activity and toxicity of three novel synthetic antibacterial agents containing *tris*(1*H*-indol-3-yl)methylium fragment were studied in vitro and in vivo. All compounds in vitro revealed high activity (minimal inhibitory concentration (MIC) 0.13–1.0 µg/mL) against bacteria that were either sensitive or resistant to antibiotics, including multidrug-resistant clinical isolates. The derivatives combining high antimicrobial activity with relatively low cytotoxicity against human donor fibroblasts HPF-hTERT were subjected to further testing on mice. In vivo they revealed fairly good tolerance and relatively low toxicity. Acute toxicity was evaluated, and the main indicators of toxicity, including LD_50_ and LD_10_, were determined. A study of compounds in vivo showed their efficiency in the model of staphylococcal sepsis in mice. The efficiency of compounds may be due to the ability of indolylmethylium salts to form pores in the cytoplasmic membrane of microbial cells and thereby facilitate the penetration of molecules into the pathogen.

## 1. Introduction

Recently, compounds containing aryldi(indolyl)methyl or *tris*(indolyl)methyl fragments have attracted the interest of researchers. This is due to the fact that some compounds of this type exhibit useful biological properties, such as antimicrobial and antiproliferative activity [1,2,3,4,5,6,7,8,9,10]. High activity against various lines of tumor cells make it possible to consider compounds of this class primarily as a basis for the development of antitumor drugs [3,4,5,6].

The situation in the field of therapy of infectious diseases has been significantly complicated due to the wide spread of pathogens resistant to known antibiotic drugs [11,12,13]. To solve this problem, we propose to try and prevent the drug resistance by the rational use of antibiotics and by development of new antimicrobial agents capable of overcoming drug resistance [14]. In previous work, we obtained a series of symmetric *N*-alkyl-substituted *tris*(indolyl)methylium salts possessing significant antibacterial and antifungal activity [15]. One of the main advantages of these compounds was their high activity against Gram-positive bacteria, including multidrug-resistant strains. The main disadvantage was their high cytotoxicity, detected in experiments with mammalian cell cultures in vitro [15].The study of structure–activity correlation in this class of compounds made it possible to identify ways of increasing the activity of new compounds as well as reducing their cytotoxic properties [16,17]. Since a crucial factor affecting both the level of antibacterial activity and the level of cytotoxicity of compounds in the series of *N*-(hydroxyalkyl) derivatives of tris(1*H*-indol-3-yl)methylium salts is the lipophilicity of the molecule, very good prospects for reducing cytotoxicity have been discovered by changing the lipophilicity of such molecules [17]. Another important approach for reducing toxicity is the development of compounds with one of the indole rings of a trisindolylmethylium salt containing maleimide as a substituent. It is known that some maleimide derivatives can suppress a number of microbial enzymes [18,19]. Combination with maleimides made it possible to reduce the cytotoxicity of tris(indolyl)methylium salts without a significant decrease intheir antimicrobial activity [20].

Here, we present the results of a detailed study of activity and cytotoxicity in experiments in vitro of several promising compounds of this class, including a number of compounds carrying a maleimide fragment with alkyl and aryl substituents. The derivatives can be divided into two groups of compounds: the ones with maleimides in their chemical structure and derivatives without it.

Compounds combining high antimicrobial activity and relatively low cytotoxicity in human cells were selected for further study in laboratory animals (mice). In vivo they have shown good tolerability and low toxicity. The main indicators of toxicity of these compounds were determined, including LD_50_ and LD_10_. The study of compounds in vivo showed their high efficiency in the model of staphylococcal sepsis in mice. We assume that the increase in the efficiency of compounds may be due to ability of the indolylmethylium fragment to form pores in the cytoplasmic membrane of microbial cells.

## 2. Results

### 2.1. Chemistry

**Figure 1:**
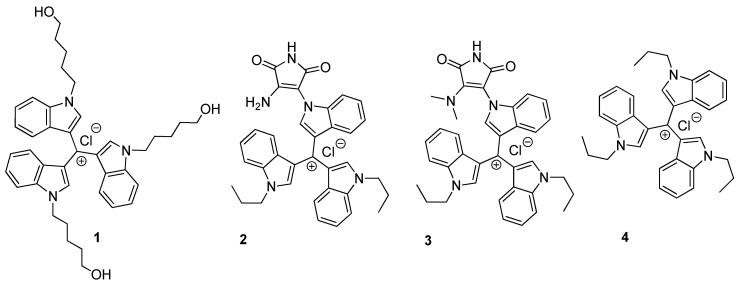
Chemical structures of compounds **1**–**4**.

The structural formulas of *tris*(1*H*-indol-3-yl)methylium derivatives represent two groups of compounds, namely, derivatives with maleimide fragment (**2**,**3**) and derivatives without maleimide fragment in their structure (**1**,**4**), as shown in Figure 1. The chemical synthesis of all the compounds has been described previously: *tris*(1-(5-Hydroxypentyl)-1*H*-indol-3-yl) methylium chloride (**1**) [17]; (1-(4-Amino-2,5-dioxo-2,5-dihydro-1*H*-pyrrol-3-yl)-1*H*-indol-3-yl)bis(1-propyl-1*H*-indol-3-yl) methylium chloride (**2**) [20]; 1-(4-Dimethylamino-2,5-dioxo-2,5-dihydro-1*H*-pyrrol-3-yl) -1*H*-indol-3-yl) bis(1-propyl-1*H*-indol-3-yl) methylium chloride (**3**) [20]; and *tris*(1-propyl-1*H*-indol-3-yl) methylium chloride (**4**) [15].

All test compounds were at least 98% pure (by HPLC). All compounds were prepared as solutions in dimethyl sulfoxide (DMSO) with the final concentrations ranging from 1.6 to 6.4 μg/mL for in vitro testing. Experiments in vivo require the special dosage forms of pharmaceutical substances, which were special injection solutions with a physiologically acceptable composition, suitable for intravenous administration in animals.

For the development of dosage forms for compounds **1**–**3**, which are quite soluble in water, various combinations of solubilizers were investigated in order to increase their solubility and stability, namely: Tween-80 (AppliChem, Darmstadt, Germany), Polyethylene glycol P-600 (Sigma-Aldrich, St. Louis, MO, USA), (2-hydroxypropyl)-β-cyclodextrin (Sigma-Aldrich), “Glucose-E” (Glucose solution for infusions 5%) and others. Using solutions in 5% isotonic glucose in water for intravenous administration (“Glucose-E”-5% glucose solution for infusions) turns out to be the most effective way of preparing compounds **1**–**3** for administration to animals. The resulting true solutions with concentration exceeding 5 mg/mL for each compound were sterilized by filtration through sterile Sterivex 0.22 μm filters (Millipore, Burlington, MA, USA) and were stable for more than 5–7 days at room temperature.

### 2.2. Biological Evaluation In Vitro: Detection of Antimicrobial Activity and Cytotoxicity

We have tested the antimicrobial activity in vitro of over 100 *tris*(indolyl)methane derivatives against 16 strains of Gram-positive, Gram-negative bacteria and fungi including either sensitive or drug-resistant strains from ATCC, as well as resistant clinical isolates from the culture collection of the Laboratory for Control of Hospital Infections (Sechenov University, Moscow, Russia). The minimum inhibitory concentrations (MICs) were determined by the microdilution method in a cation-adjusted Müller–Hinton medium for bacteria and in a liquid culture medium RPMI 1640 with L-glutamine without sodium bicarbonate for fungi in accordance with the requirements of the Institute of Clinical and Laboratory Standards (CLSI/NCCLS) [21,22,23]. More than 70% derivatives demonstrated high activity against Gram-positive bacteria, including collection cultures and multidrug-resistant clinical isolates. The MICs of 20 of the most active derivatives against Gram-positive organisms ranged from 0.13 to 2.0 μg/mL. As a rule, their activity against Gram-negative bacteria was usually not that high. The antimicrobial activity of derivatives **2** and **3**, which are indolylmethylium salts possessing a maleimide fragment and a derivative **1** without such a fragment, is shown in Table 1. For comparison, the antimicrobial activity of prototype **4** is shown. The antibacterial antibiotic levofloxacin and the antifungal amphotericin B were used as reference drugs (Table 1).

The highest level of antibacterial activity, especially against Gram-positive bacteria (MIC 0.13 μg/mL), was demonstrated by compounds **2** and **3,** which possessed maleimide fragments, and by compound **4** (MIC 0.25 μg/mL), a *tris*(indolyl)methylium salt with C3 alkyl radicals, which was taken as a reference. Their activity against Gram-positive bacteria was comparable with levofloxacin or even better.

Compound **2** (MIC 0.5 μg/mL) was highly active. Compound **1**, which is *N*-(Hydroxyalkyl) derivative of *tris*(1*H*-indol-3-yl)methylium salt, was somewhat inferior (MIC 2.0 μg/mL). Compounds **1** and **3**, which are highly active against Gram-positive bacteria, showed some activity against Gram-negative bacteria too.

Compounds **3** and **4** possess a pronounced activity against yeast (*Candida albicans*) and fungi (*Aspergillus niger*) (MIC 2.0 μg/mL). Compound **2** revealed low antifungal activity. Compound **1** had no antifungal activity at all.

In general, it should be recognized that all the compounds listed in Table 1 were equally active against both sensitive and resistant bacteria, including strains with multidrug resistance. For example, all compounds showed high activity not only against *Staphylococcus aureus* ATCC 25923 and clinical isolate *S. aureus* 10, which are antibiotic-sensitive, but also against two methicillin-resistant strains (MRSA) (*S. aureus* 5 and *S. aureus* 100KC). They were also active against *S. aureus* ATCC 3798, which is resistant to ampicillin, oxacillin, cefuroxime and carbenicillin (antibiotics of penicillin and cephalosporin groups), as well as to clindamycin, erythromycin, rifampicin, ciprofloxacin and levofloxacin. All compounds were active against *S. aureus* ATCC 700699, which is resistant to levofloxacin, and the *Staphylococcus epidermidis* 533 strain, which is resistant to gentamicin.

At the same time, there were some differences. For example, compound **3** was not active against the clinical isolate *Staphylococcus haemoliticus* 585, which is resistant to penicillins, aminoglycosides and erythromycin. It was also inactive against *Enterococcus faecium* 569, which possesses resistance to cefuroxime, clindamycin, gentamycin, vancomycin and doxycycline. Against the latter strain compound **1** revealed reduced activity.

The cytotoxic properties of the obtained compounds were tested in vitro with the use of the MTT assay as described previously [17] on the donor (postnatal) human fibroblasts immortalized by transfection of the hTERT gene of the catalytic component of telomerase (hereinafter HPF-hTERT) to select derivatives possessing relatively low cytotoxicity.

In terms of the in vitro cytotoxic action against HPF-hTERT, the studied compounds differed significantly. The prototype compound **4**, which possesses extremely high antimicrobial activity, exhibited the highest level of cytotoxicity with its IC_50_ 0.07 μg/mL. The cytotoxicity of the new derivatives was not as pronounced. For example, the IC_50_ values for compounds **3** and **2** were 0.57 and 2.77 μg/mL, respectively. The IC_50_ values for derivative **1** were even higher. According to the degree of reduction of cytotoxicity, the studied compounds could be arranged in the following order: **1** < **2** < **3** < **4**, where the least cytotoxicity (IC_50_ 12.6 μg/mL) was demonstrated by compound **1**.

In general, compared with precursor compound **4**, the new derivatives with high antimicrobial activity show a significant reduction in cytotoxicity. The best ratio of antimicrobial activity to cytotoxicity was shown by compound **1** as well as the maleimide-derived compounds (**2**,**3**). In some cases, the introduction of a maleimide fragment into the trisndolylmethane structure even made it possible to slightly increase the antimicrobial activity of compounds. For example, compound **3** had increased activity against Gram-positive bacteria compared with its predecessor, **4**.

Active derivatives based on a maleimide fragment (compounds **2**, **3**) with reduced cytotoxicity, as well as compound **1,** were selected for further investigation in experiments in vivo.

### 2.3. Biological Evaluation In Vivo

#### 2.3.1. Toxicity Studies in Animals (Mice)

Toxicological studies made it possible to calculate the main doses characterizing the toxicity of the studied compounds: the tolerable, the toxic and the lethal. The reasons for the death of animals were determined. Determination of tolerated doses (TD) is necessary for conducting further detailed testing of the most promising compounds in order to reveal their ability to suppress experimental bacterial sepsis in mice.

In toxicological experiments with each compound, 72 to 108 animals were used and divided into 8 or 9 experimental groups (6 to 12 mice each), in which the animals received various doses of the tested compounds. There was also a control group of animals, which was injected with the corresponding solvent instead of the experimental compound, and a control group of intact animals. The prepared substances were injected into the animals intravenously (into the tail vein); the dose range was from 1.0 to 100 mg/kg.

The lethal doses of the drugs that caused the death of more than 50% of the animals in the group in the first minutes and hours after administration differed and amounted to 35–50 mg/kg for compound **2** and 25–40 mg/kg for compound **3**.

When testing compound **3**, the death of animals was observed during the first day after administration, and for compound **2**, deaths began just in the first two hours after administration.

The test on drug **1** showed that for this compound the lethal dose, which causes the death of more than 50% of the animals, is in the range from 26 to 30 mg/kg. However, the death of animals was observed only within the first 15 min after drug administration.

Animals that received lethal doses of drugs and died in the first minutes after the administration rapidly developed tachypnea, followed by abnormal Cheyne–Stokes respiration and then tonic convulsions, and the animals assumed a lateral position while respiration and cardiac activity ceased. At high doses, neurotoxicity was the cause of death.

Lower doses also led to the death of the animals, albeit later, and the animals became lethargic and assumed a lateral position. Autopsy: the heart was in systole, the lungs were full-blooded, the mucous membrane of the abdominal cavity and intestines were pale pink, and the vessels were dilated and filled with blood. There were no hemorrhages or effusion into the abdominal cavity.

The main calculated doses characterizing the toxicity of the studied compounds, determined with (http://www.analystsoft.com/, accessed on 11 January 2022), are presented in Table 2.

Surviving animals were euthanized with diethyl ether on day 30. Autopsies of animals subjected to planned euthanasia did not reveal any pathological macroscopic changes, except for signs of rapid death.

Corpses of mice were of normal constitution. The coat was shiny, dry and clean. Natural holes were without any peculiarities. Visible mucous membranes were pale and cyanotic. Autopsy: the peritoneum, pleura and pericardium were smooth and shiny. There was no free fluid in the abdominal cavity, pericardial cavity or pleural cavities.

The heart was in diastole. The cavities were not dilated and contained dark-red blood coagulation. The thymus was yellowish-whitish and moderately dense. The lungs were airy, pale pink in color and evenly colored. A small amount of foamy, transparent liquid was released on the cut.

The kidneys were dense and a dark-claret color. The capsule was smooth, shiny and easily removable, revealing a smooth surface. On the cut, the cortical and medullary layers were clearly distinguishable. The ovaries were not enlarged; the surface was bumpy, with a moderate amount of *corpus luteum*. The liver was dense; the surface wasdark burgundy, smooth and shiny. Upon cutting, the tissue looked to be filled with normal brown, homogeneous blood.

The pancreas was not enlarged and of a doughy consistency. The spleen was not enlarged and dense, and the capsule was smooth and shiny. Upon cutting, without scraping the pulp, the tissue was dark cherry and homogeneous.

The mass coefficients of the internal organs of mice that survived the intravenous administration of drugs in a dose between LD_16_ and LD_84_ did not change in comparison with the mass of the organs of animals in the control group.

The absence of changes in internal organs in the body of animals suggests that in tolerated doses the changes that occur upon acute intoxication are reversible. Most of the tested drugs have thus proven to be promising and require further study.

Thus, the study of toxic indicators and the identification of the maximum tolerated dose (MTD) corresponding to LD_10_ allowed us to proceed to further trials of drugs to determine their effectiveness in a model of staphylococcal sepsis in mice.

#### 2.3.2. Efficiency in the Model of Staphylococcal Sepsis in Mice

The results of an experimental study of compounds **1**, **2** and **3** are presented in Table 3. To determine the ability of drugs to suppress staphylococcal sepsis, a clinical isolate of *S. aureus* 10, lacking drug resistance and adapted for use in animals, was used. Animals, divided into several experimental groups, were intravenously infected with a lethal dose of *S. aureus* (0.1 mL each) (Groups 1–5). One hour after infection, the animals of experimental groups 1–4 were intravenously injected once with the test preparations in various doses (0.2 mL each). Along with the testing of compounds of the trisndolylmethylium class, a similar experiment was carried out with levofloxacin, taken as a reference drug for a separate group of animals (Group 4). The control group of animals received an intravenous injection of 5% glucose solution instead of the tested preparations (Group 5). Another control group, which also received a single injection of 5% glucose solution, consisted of animals that were not exposed to infection (Group 6). To study each drug, 110–150 animals were required, which were divided into small subgroups of 10 mice each to test various doses of the compounds studied. The number of dead animals and the time of their death were recorded daily, and the appearance of the animals and their weight were assessed. The duration of the experiment was 14 days.

As evidenced by the results presented in Table 3, most of the tested compounds showed good tolerability and pronounced efficacy in suppressing staphylococcal sepsis in mice.

Mice in the control group were infected with a lethal dose of *S. aureus* (8 × 10^8^ CFU/mouse) and had no treatment (Group 5); the first death occurred on the 3rd day of the experiment and by the 9th day all the animals of this control group died. No death was observed in the group of intact animals not infected with staphylococcus (Group 6).

Administration of the test preparations to infected animals resulted in a delay and decrease in the death of animals. In the experimental group 3, receiving drug **3**, the first mortality was observed later—on the 6–7th day of the experiment. The last death occurred on day 12.

When drug **3** was administered at a dose of 2 mg/kg, a 100% survival rate was observed. When drug **2** was administered, 100% survival was not observed in any of the experimental subgroups. The maximum survival rate with the administration of drug **2** was 80% and was observed in a subgroup of animals that received the drug at a dose of 30 mg/kg (Group 2).

Using the StatPlus 2006 professional 3.8.0 program (http://www.analystsoft.com/, accessed on 11 January 2022) the ED_50_ values were determined for both drugs. The ED_50_ value for compound **2** was 18.27 (11.9 ÷ 24.95) mg/kg and 1.09 (0.77 ÷ 1.42) mg/kg for drug **3**.

The antibiotic levofloxacin (Group 4) used as a reference drug under similar conditions (single injection) saved the lives of 50% of infected animals at about 4 mg/kg. In our experiments the ED_50_ index for levofloxacin was 3.5 (2.85 ÷ 4.15) mg/kg.

The study of compound **1** (Group 1) showed that it also protects mice from staphylococcal infection, although it provides a much weaker defense. Compound **1** was tested at doses of 3–16 mg/kg. The survival rate of animals receiving the drug at a dose of 9–11 mg/kg barely reached 40%, and with an increase in the dose, a decrease in survival rate occurred, due to the manifestation of the toxic properties of the compound itself. The ED_50_ for the drug **1** for a single administration, determined using the StatPlus 2006 professional 3.8.0, was 7.6 (4.85 ÷ 11.45) mg/kg.

In mice receiving compounds **2** and **3** the dynamics of changes in body weight during the observation period werecharacterized by an initial sharp decrease in weight and its subsequent slow recovery compared to the control group of untreated animals. Mice treated with drug **1** also lost weight, but the weight recovery of the surviving animals was much slower.

Repeated administration of drug **1** at a relatively low dose (3 mg/kg) significantly increased its efficacy (Table 4). While with a single administration of drug **1** at a dose of 3 mg/kg, 12.5% survived (Group 1), the use of repeated administration of the drug on the next day (at the same dose) led to a doubling of the number of surviving animals (Group 2). Additional administration of the drug on the 3rd day of the experiment led to a further increase in the survival rate to 37.5% (Group 3). All infected animals that received a 5% glucose solution instead of drug **1** (Group 4) died as early as on the 9th day of the experiment. These results indicate that drug **1** may be promising if administered repeatedly.

A quantitative characteristic of the width of the therapeutic action of a drug is its therapeutic index (TI), which is the ratio of LD_50_ to ED_50_ [24]. The therapeutic index of the tested drugs varied (Table 5). For compounds **1** and **2** it was 3.45 and 2.3, respectively. For compound **3** its value was even greater at 22.2.

The best TI value was noted for compound **3**, which was somewhat inferior to its analogs in terms of cytotoxicity in experimentsin vitro. Apparently, there are serious differences in the effect of compounds on cell cultures in vitro and in their effect in vivo when various transformations occur in the animal organism, associated with the pharmacokinetic and pharmacodynamic properties of the drug. Thus, the combined therapeutic parameters of the new compounds **1**, **2** and **3** indicate their good therapeutic efficacy, especially for drug **3**, and, accordingly, the prospects for their further study in order to create new highly effective antimicrobial drugs.

## 3. Discussion

Modification of the natural antibiotic turbomycin A, primarily on pyrrole fragments of indole rings, made it possible to obtain derivatives that significantly exceed the natural antibiotic, both in terms of the level and spectrum of antimicrobial activity [15,17,20].

At the same time, the first obtained derivatives were characterized by a high cytotoxicity [2], which makes it difficult to use them as effective antimicrobial drugs. Conducting modifications, we obtained compounds containing a maleimide fragment in their structure [20], which allowed us to overcome this drawback and significantly reduce the toxic action of compounds on human cells. At the same time, we managed to maintain a high level of antibacterial activity, mainly against Gram-positive bacteria. All the active derivatives were active against strains resistant to most currently used drugs as well, including multidrug-resistant (MDR) strains.

New highly efficient methods of synthesis have been developed [15,17,20]. Prototypes of dosage forms have been developed, and promising compounds have been studied in vivo experiments using laboratory animals (mice). The obtained compounds were shown to be well tolerated when administered intravenously. The main indicators of toxicity have been identified. The ability of the studied compounds to suppress experimental staphylococcal sepsis in mice was shown.

A promising direction is the development of *tris*(indolyl)methylium salts containing maleimide moiety [20]. The latter are known for their ability to suppress various enzymes, including various protein kinases [11,12,13,14], and function as active inhibitors of intracellular signaling [25]. Derivatives of *tris*(indolyl)methane, for their part, possess the ability to actively form ion channels in the structure of cell membranes and can easily penetrate the microbial cell; thus, they can be considered as carriers, increasing penetration of a target molecule into the microbial cell.

In general, the results obtained demonstrate the possibility of creating new highly effective antimicrobial drugs based on *tris*(indolyl)methylium salts, including compounds containing a maleimide fragment.

## 4. Materials and Methods

### 4.1. Preparation and Purification of New Compounds, Their Physical and Chemical Characteristics, Preparation of Solutions

The synthesis of compounds and the scheme of their preparation and purification, as well as physicochemical characteristics, were described earlier [15,17,20]. To evaluate the biological activity in vitro, the compounds were dissolved in DMSO at a concentration of 2.0–6.4 mg/mL, followed by preparation of two-fold dilutions in the same solvent, if necessary. The solutions were further diluted in an aqueous medium for the final concentration of DMSO not to exceed 1%, and the concentration of the compounds ranged from 64.0 to 0.13 μg/mL. To study compounds in vivo, solutions for intravenous administration in 5% glucose solution were prepared. Solutions were sterilized by filtration through 0.22 µm filters (Millipore, Burlington, MA, USA).

### 4.2. Microbial Strains, Nutrient Media, Cultivation Conditions

Strains of Gram-positive, Gram-negative bacteria and fungi including sensitive or drug-resistant strains from ATCC, as well as resistant clinical isolates from the culture collection of the Laboratory for Control of Hospital Infections (Sechenov University, Moscow, Russia), were used. Clinical isolates of Gram-positive bacteria *Staphylococcus aureus* 5 (MRSA), *Staphylococcus aureus* 10, *Staphylococcus aureus* 100KC (MRSA), *Staphylococcus epidermidis* 533, *Staphylococcus haemoliticus* 585 and *Enterococcus faecium* 569, as well as collection cultures of Gram-positive bacteria *Staphylococcus aureus* ATCC 700699, *Staphylococcus aureus* ATCC 25923, *Staphylococcus aureus* ATCC 3798 and collection cultures of Gram-negative bacteria *Escherichia coli* ATCC 25922, *Klebsiella pneumoniae* ATCC 13883, *Proteus vulgaris* ATCC 13315, *Salmonella cholerasuis* ATCC 14028, *Pseudomonas aeruginosa* ATCC 27853 and collection cultures of the yeast *Candida albicans* ATCC 14053 and the fungi *Aspergillus niger* ATCC 16404 were used. To grow the microorganisms, we used Trypticase Soy Agar (TSA, Sigma-Aldrich Co., Burlington, MA, USA). Trypticase Soy Agar, BBL, was used for cultivation of *Staphylococcus* spp. and *E. coli*. For cultivation of *Enterococcus* spp. and *P. aeruginosa* we used a ready-made dry medium Columbia Agar Base, BBL. The media were sterilized by autoclaving at 121 °C for 15 min. Yeast culture *C. albicans* was grown on Sabouraud agar (peptone—10 g, glucose—40 g, agar—20 g, distilled water—1 L, pH 6.0), and filamentous fungi *A. niger* was grown on potato–glucose agar (potatoes—200 g, glucose—20 g, agar—15 g, distilled water—1 L, pH 5.5–6.0).

To determine the antibacterial effect, a liquid Müller–Hinton medium (Becton, Dickinson and Company, Cockeysville, MD, USA) was used; to determine the effect of compounds on fungi we used RPMI 1640 medium with L-glutamine, without sodium bicarbonate, which was prepared from a dry medium (ICN Biomedicals Inc., Costa Mesa, CA, USA) by dilution in distilled water, subsequent buffering with 0.165 M of morpholinepropanesulfonic acid (MOPS, Acros Organics B.V.B.A., Morris Plains, NJ, USA) and adjusting the pH of the medium to 7.0 by adding 1 N of NaOH solution. The culture medium RPMI 1640 was sterilized by pressure filtration through Sterivex-GV 0.22 μm filters (Millipore, Burlington, MA, USA).

### 4.3. Estimation of the Antibacterial and Antifungal Activities In Vitro

The minimum inhibitory concentrations (MIC) for Gram-positive and Gram-negative bacteria were determined by two-fold serial microdilution method in a cation-adjusted Müller–Hinton medium for bacteria and in a liquid culture medium RPMI 1640 with L-glutamine without sodium bicarbonate for fungi in accordance with the requirements of the Institute of Clinical and Laboratory Standards (CLSI/NCCLS) [21,22,23]. The antibacterial antibiotic levofloxacin and the antifungal antibiotic amphotericin B from Sigma-Aldrich were used as reference drugs.

### 4.4. The Cytotoxic Activity

The cytotoxic activity of the compounds was evaluated on the healthy donor (postnatal) human fibroblasts immortalized by transfection of the hTERT gene of the catalytic component of telomerase (hereinafter HPF-hTERT) that were obtained from N.N. Blokhin Russian Cancer Research Center. HFC-hTERT cells were cultured in Dulbecco’s modified medium (DMEM) containing 2 mM of L-glutamine, 10% fetal calf serum, 100 U/mL of penicillin and 100 μg/mL of streptomycin. Incubation was performed in a humidified atmosphere of 5% CO_2_ at 37 °C. In the experiment, we used cells in the logarithmic growth phase. To study the cytotoxicity of compounds, 190 μL of cell suspension (~2 thousand cells per well) was added to the wells of 96-well plates and incubated for 16 h. On the day of the experiment, serial dilutions of the test compounds were prepared in DMSO (stock solution—10 mM in 100% DMSO). Further dilutions were conducted in a nutrient medium. The final concentrations of compounds in the experiment were in a concentration range from 0.0025 to 50 μM. The final concentration of DMSO was 1% or less. Each concentration was presented in 3 repetitions. The wells that did not contain the tested preparations served as a control. The cells were incubated with the test compounds for 72 h. After the end of incubation, 20 μL of MTT solution in growth medium (5 mg/mL) was added to the wells. The cells were incubated for 2 h until a purple color developed, after which the culture medium was removed. DMSO was added to the wells, and the sediment was suspended. Optical density was measured with a Thermo Scientific Multiscan FC spectrophotometer at 570 nm. The values of optical density in wells without addition of test compounds (control) were taken as 100% cell survival. The percentage of survival of cells incubated with the test compounds was calculated by dividing the optical density in the corresponding well (average of 3 independent measurements) by the optical density in the control. The concentration of drugs causing 50% cell death (IC_50_) was evaluated and expressed in μg/mL. The average inhibiting doses required for the compounds to block the viability of 50% of cells (IC_50_) are given in Table 1.

### 4.5. Determination of the Acute Toxicity and Antimicrobial Activity of the Tested Compounds In Vivo

All experiments in vivo were performed in accordance with the European Convention for the Protection of Vertebrate Animals [26] and the national standard of the Russian Federation R 53434-2009 «Good Laboratory Practice» [27].

The study was conducted on adult female SHK mice (18–20 g) obtained from the animal breeding unit of the FMBA (Scientific Center for Biomedical Technologies of the Federal Biomedical Agency, Moscow, Russia). The animals were randomly divided into groups (*n* = 6 for the study of acute toxicity, *n* = 10 (or 8) to study the efficacy of suppressing staphylococcal sepsis). The study of the effectiveness of drugs in the treatment of staphylococcal sepsis required the use of 110–175 mice for each drug, 10 animals per group. The animal study was approved by the Ethics of Animal Experimentation of Gause Institute of New Antibiotics (protocol No 13/2019).

At the beginning of work, mice at the age of 8 weeks had a body weight of 18–20 g and acclimatized to the new conditions of detention for 14 days before the start of the experiment. During this period, a daily examination of the external condition of the animals was conducted. The selected mice for experimental groups were animals without signs of deviations in appearance and chosen at random, so that the individual value of the mass deviated from the mean by no more than 10%. Each animal was assigned an individual number, marked with a puncture of the auricle and fixed on the cage card. Groups of mice were formed with 6 or 10 animals in each group.

The animals were kept in polycarbonate cages (6 or 10 animals in each) on bedding. As bedding we used certified sterilized dust-free sawdust supplied by Laboratorkorm Association (Moscow). The standard pelleted food “Food for the maintenance of laboratory rodents”, supplied by Laboratorkorm, was fed into the aft recess of the steel grating cover of the cage. Tap water, or“Drinking water” (GOST 2874-82), was given ad libitum in standard autoclaved drinking bottles with steel spout lids. The animals were kept in controlled environmental conditions: 18–22 °C and 50–65% relative humidity. Temperature and humidity were monitored using an air conditioner. In the animal rooms, a 12 h lighting cycle and 8–10-fold change in air volume per hour were maintained.

### 4.6. Preparations for Intravenous Administration

To prepare solutions of preparations suitable for intravenous administration, dried test compounds with a purity of at least 99% were dissolved in a 5% glucose solution for intravenous administration at a concentration of 3.2 or 6.4 mg/mL. The solutions were sterilized by filtration through Sterivex 0.22 μm filters (Millipore, Burlington, MA, USA). Sterile solutions of drugs with a reduced concentration were then prepared, which allowed for the introduction ofvarious doses of drugs with the same volume (0.2 mL) of solutions. The test preparations were stored at a temperature no higher than 25 °C.

### 4.7. Acute Toxicity Study

The study of acute toxicity of each drug was conducted on 72 mice (6 mice per group) using a solution of the test drug prepared in 5% glucose solution. The drug was injected into the tail vein of the animals (0.2 mL) using a syringe with a metal needle in a dose range of 1.0 mg/kg to 70 mg/kg.

From the first day after administration, the animals were under continuous observation. The number of dead animals and the time of their death were recorded daily. The body weight of the animals and their food consumption was recorded once a week, and the clinical signs of the animals’ health were assessed daily. The duration of observation of the animals was at least 30 days after the last death of the animal. Doses characterizing the toxicity of drugs were calculated by the Litchfield and Wilcoxon method using the StatPlus 2006 software AnalystSoft StatPlus—statistical analysis program, version 2006 (http://www.analystsoft.com/, accessed 11 January 2022). The mean and standard deviation were calculated for all data. The differences are defined as significant at *p* ≤ 0.05.

### 4.8. Study of the Drug Efficacy in the Treatment of Staphylococcal Sepsis in Mice

The experiment used a strain of *S. aureus* 10, having no drug resistance and adapted for use in animals. Initially, the 100% lethal dose (LD_100_) was determined for this pathogen strain in SHK mice, i.e., the dose of the pathogen at which the death of 100% of the animals was recorded. LD_100_ dose of staphylococcus detected in the experiment was 8.0 × 10^8^ CFU/mouse, which was then used for infecting animals.

### 4.9. Experiment Protocol

The chemotherapeutic efficacy of *tris*(indolyl)methane derivatives was determined in a model of staphylococcal sepsis in mice. Mice of the SHK colony, weighing 18–20 g, were infected intravenously (into the tail vein) with a lethal dose of staphylococcus (0.1 mL of solution). One hour after infection, mice were injected intravenously (0.2 mL of solution each) with the tested tris(indolyl)methane derivatives in the range of doses from 0.1 to 40 mg/kg. For tested drugs, we used solutions prepared in 5% glucose solution. The study of the effectiveness of each drug was conducted on 115–155 mice (10 mice per group). The duration of the experiment was 14 days. The number of dead animals and the time of their death were recorded daily, and the body weight of the animals was recorded three times a week. Their feed intake was monitored, and clinical signs of animal health were assessed daily.

To determine the effectiveness of the tested drugs, 50% effective doses (ED_50_) were used, which was determined by the Behrens method (frequency accumulation method), as well as by the Litchfield and Wilcoxon method using the StatPlus 2006 software. For all data, the mean and standard deviation were calculated. The differences were defined as significant at *p* ≤ 0.05.

## 5. Conclusions

An in vitro investigation demonstrated that several novel *tris*(indolyl)methylium derivatives, including compounds with maleimide fragment in their structure, possess high antibacterial activity against Gram-positive bacteria and the strains with multiple-drug resistance, and at the same time, they showed low toxicity on human cells. After further investigation in vivo, they revealed good tolerance and relatively low toxicity and showed high efficiency in the model of staphylococcal sepsis in mice. The obtained results demonstrate that *tris*(indolyl)methylium derivatives, some of them containing a maleimide fragment in their structure, could be a good base for the development of new, highly effective antimicrobial drugs.

## Figures and Tables

**Table 1:** Antimicrobial activity of compounds in vitro.

Compounds	MIC, μg/mL				
	Control	1	2	3	4
Bacterial Strains	Gram-Positive Bacteria				
*Staphylococcus aureus* ATCC 25923	0.25 (Lf)	2	0.5	0.13	0.25
*Staphylococcus aureus* ATCC 3798	32 (Lf)	2	0.25	0.13	0.25
*Staphylococcus aureus* 100KC	32 (Lf)	2	0.5	0.13	0.5
*Staphylococcus aureus* ATCC 700699	16 (Lf)	2	0.5	0.13	0.25
*Staphylococcus aureus* 10	0.13 (Lf)	1	0.5	0.25	0.25
*Staphylococcus aureus* 5	0.25 (Lf)	4	0.25	0.5	0.5
*Staphylococcus epidermidis* 533	0.5 (Lf)	1	0.5	0.5	0.13
*Staphylococcus haemoliticus* 585	0.5 (Lf)	4	2	>64	0.5
*Enterococcus faecium* 569	1 (Lf)	8	8	>64	1
Bacterial Strains	Gram-Negative Bacteria				
*Escherichia coli* ATCC 25922	0.06 (Lf)	8	>64	16	32
*Klebsiella pneumoniae* ATCC 13883	0.25 (Lf)	2	>64	>64	>64
*Proteus vulgaris* ATCC 13315	4 (Lf)	64	16	>64	1
*Salmonella cholerasuis* ATCC 14028	0.13 (Lf)	>64	>64	>64	32
*Pseudomonas aeruginosa* ATCC 27853	1 (Lf)	>64	32	16	64
Fungi Strains	Fungi				
*Candida albicans* ATCC 14053	1 (Am B)	>64	12	2	1
*Aspergillus niger* ATCC 16404	1 (Am B)	>64	8	2	2
Test Cells	Cytotoxic Activity IC_50_, μg/mL				
HPF-hTERT	>50 (Lf); 0.7 (Am B)	13	2.8	0.6	0.07

Lf—levofloxacin; Am B—amphotericin B; IC_50_—concentration of compound inhibiting the growth of cells by 50%.

**Table 2:** Doses that characterize the toxicity of various compounds based on indolylmethylium salts.

	Doses, mg/kg		
Compounds	1	2	3
LD_50_	26.2 (24.3 ÷ 28.1)	41.8 (37.6 ÷ 45.9)	24.2 (20.2 ÷ 28.2)
MTD (LD_10_)	22.6 (21.0 ÷ 24.2)	34.1 (31.0 ÷ 35.8)	16.9 (15.4 ÷ 17.6)
LD_16_	23.4	35.8	18.5
LD_84_	28.9	47.7	29.8
LD_100_	30.2	50.7	32.6

**Table 3:** Determination of the efficacy of compounds **1**, **2** and **3** in comparison with levofloxacin in a model of staphylococcal sepsis in mice.

Compound	Dose of the Drug, mg/kg	Death (%)	Survival Rate (%)
**1**	3.0	87.5	12.5
	5.0	75	25
	7.0	75	25
	9.0	62.5	37.5
	11.0	62.5	37.5
	13.0	75	25
**2**	5.0	80	20
	10.0	70	30
	15.0	60	40
	20.0	40	60
	25.0	40	60
	30.0	20	80
	35.0	30	70
**3**	0.5	70	30
	1.0	50	50
	1.5	40	60
	1.75	10	90
	2.0	0	100
Levofloxacin	1.0	90	10
	2.0	60	40
	4.0	40	60
	6.0	30	70
Control (infected mice without treatment).	-	100	0
Intact Animals	-	0	100

**Table 4:** Determination of the effectiveness in the model of staphylococcal sepsis in mice after repeated administration of drug **1** at a dose of 3 mg/kg.

Group Number	Regimen of Drug Administration	Days of Experiment (Numerator—Dead Animals, Denominator—Survivors)										Death (%)	Survival Rate (%)
		0	3	4	6	7	8	9	10	12	14		
1	One-time	0/8	1/7	2/6	4/4	5/3	5/3	6/2	7/1	7/1	7/1	87.5	12.5
2	Double	0/8	0/8	1/7	3/5	4/4	4/4	5/3	6/2	6/2	6/2	75	25
3	Three-fold	0/8	0/8	1/7	2/6	3/5	3/5	4/4	5/3	5/3	5/3	62.5	37.5
4	Control	0/8	2/6	3/5	5/3	6/2	7/1	8/0	8/0	8/0	8/0	100	0

**Table 5:** Main indicators of the effectiveness of drugs evaluated in the model of staphylococcal sepsis in mice.

	Compound		
	1	2	3
ED_50_, mg/kg	7.6 (4.85 ÷ 11/45)	18.27 (11.9 ÷ 24.95)	1.09 (0.77 ÷ 1.42)
LD_50_, mg/kg	26,2 (24.3 ÷ 28.1)	41.8 (37.6 ÷ 45.9)	24.2 (20.2 ÷ 28.2)
TI	3.45	2.3	22.2

## Data Availability

Data is contained within the article.
